# The effect of jet-lag on serum concentrations of thyroid stimulating hormone and prolactin: A case report

**DOI:** 10.11613/BM.2020.011003

**Published:** 2020-02-15

**Authors:** Merve Sibel Gungoren, Deniz Ilhan Topcu, Cevdet Zungun

**Affiliations:** 1 Duzen Laboratories Group, Ankara, Turkey; 2 Baskent University Medical Faculty, Biochemistry Department, Ankara, Turkey

**Keywords:** preanalytical phase, case report, jet-lag, thyroid stimulating hormone, prolactin

## Abstract

**Introduction:**

This case report is about the importance of sleeping status for analysis of thyroid hormone stimulating hormone (TSH) and prolactin (PRL) which arose from discordant results of a patient who was referred for serum TSH and PRL testing within 12-hour period after an intercontinental flight.

**Case description:**

An adult male patient was admitted to our laboratory for serum TSH and PRL tests and came back questioning the accuracy of his previous results.

**Further investigations:**

A new analysis with a new sample was offered. His new results were not consistent with his previous results.

**What happened:**

It was revealed that the night before the first sampling, he travelled back to Turkey from The United States of America and came to testing within 12 hours after the arrival.

**Discussion:**

Sleeping status is one of the factors that can affect laboratory results. Intercontinental flights causing jet-lag can alter the secretions of TSH and PRL which are predominantly modulated by thyrotropin-releasing hormone (TRH).

**Main lesson:**

Travel history and sleeping status are important factors to be evaluated prior sampling for hormone analysis. Patients must be informed about the importance of sampling timing.

## Introduction

Preanalytical factors include conditions dependent on both laboratory and patient (*1*). It is considerably easier for laboratory managers to control factors belonging to the laboratory to prevent preanalytical errors. However, to manage the preanalytical phase successfully, all preanalytical factors related with the patient should also be thoroughly interrogated. The most commonly asked questions related to patient conditions are nutritional status (fasting/non-fasting), smoking, pregnancy, use of medications/supplements, consumption of alcohol and exercise (*2*). A patient’s sleeping status is one of the factors that can affect various laboratory results. At the same time, it is one of the most overlooked preanalytical conditions that has to be actually known prior to testing. Information about sleeping status includes sleep deprivation (if present), frequency of sleep cycle, duration of sleep and time of waking up. The sleeping status may deviate from normal in cases of some pathological conditions (insomnia, polysomnia, *etc.*) and use of drugs disturbing sleeping habits and travelling, especially intercontinental flights. Intercontinental flights may lead to a sleep disturbance called as flight dysrhythmia, also known as jet-lag (*3*). Hormones of the central nervous system (CNS) which are released from the hypothalamus, pituitary and pineal gland can be significantly altered by jet-lag. This case report is about the importance of timing and patient’s metabolic status for hormone testing which arose from discordant serum TSH and PRL results of a patient who was referred to sampling within 12-hour period after an intercontinental flight.

## Case report and laboratory analyses

A male patient who was under routine medical follow-up was referred to our laboratory. He had no known diagnosis. He was admitted to our laboratory for serum TSH and PRL tests on a Monday morning (day 1). A serum sample was collected from the patient. His serum TSH and PRL concentrations were above the reference interval. On Saturday (day 6), he returned to our laboratory questioning the accuracy of his results. He stated that he went to another hospital on Thursday morning (day 4) and have discrepant results of serum TSH and PRL. Moreover, previous results were not compliant with his clinical condition. However, concentrations of free tri-iodothyronine (fT_3_) and free thyroxine (fT_4_) from both laboratories were consistent with each other. The laboratory of the other hospital was using the same instrument (Roche Cobas e601 immunoassay analyser, Mannheim, Germany) and reference intervals (0.3 – 4.0 µIU/mL for TSH and 3.0 – 14.7 ng/mL for PRL).

## Further investigations

We explained that the samples were not the same and asked for any difference in his conditions. He stated no remarkable change in his lifestyle, *etc.* We offered him another analysis. All test results can be found in Table 1. All samples were collected in the morning, around 9 a.m.

**Table 1:** Laboratory results of the patient

**Parameter, unit**	**Reference interval**	**Day 1** **(Düzen Laboratory)**	**Day 4** **(Other Laboratory)**	**Day 6** **(Düzen Laboratory)**
TSH, µIU/mL	0.3 – 4.0	9.1	3.0	2.8
PRL, ng/mL	3.0 – 14.7	16.3	9.0	8.7
fT_3_, pmol/L	3.1 – 6.8	4.8	4.5	4.4
fT_4_, pmol/L	12 – 22	15.8	15.7	15.4
TSH – thyroid-stimulating hormone. PRL – prolactin. fT_3_ – free tri-iodothyronin. fT_4_ – free thyroxine.				

## What happened?

**Figure 1:**
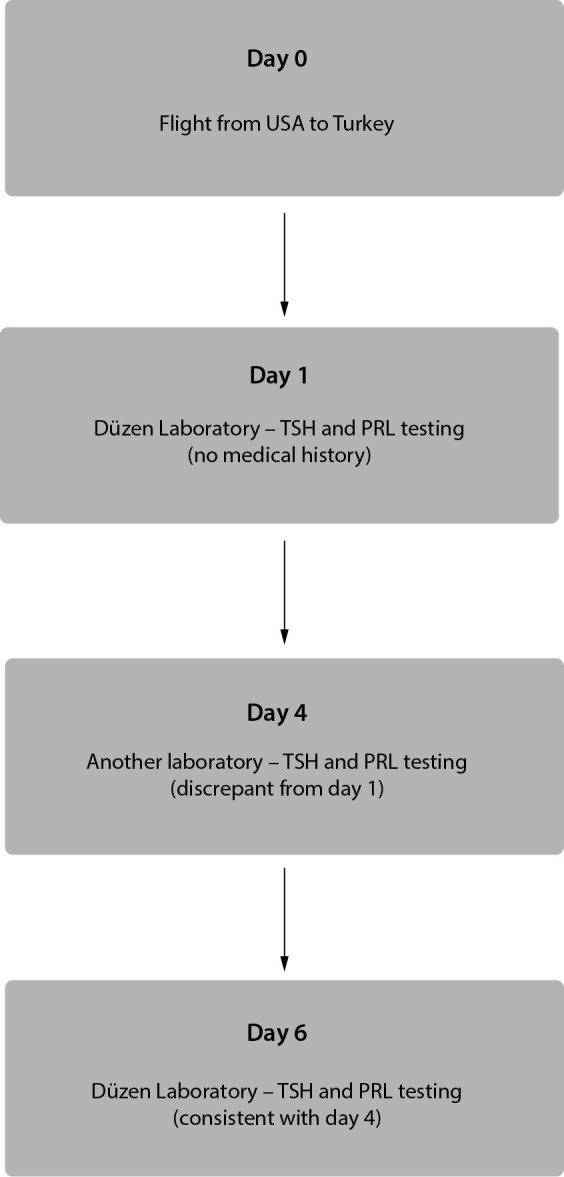
Timeline of events. TSH – thyroid-stimulating hormone. PRL – prolactin.

We persistently tried to interrogate him about the differences between conditions prior to sampling. We asked about exercise, resting, and any remarkable activity before sampling such as travelling. He confessed that the night before first sampling (day 0), he travelled back to Turkey from The United States of America (USA) and came to testing within 12 hours after the arrival. The timeline of events can be found in Figure 1. During the interview, it has been revealed that he had not been informed about the timing and prerequisite conditions for sampling. Although his doctor ordered these tests previously, he had decided to go to testing right after his travel by himself. It has been explained to him that intercontinental flight may have a significant impact on CNS hormones.

## Discussion

We report a case which arose from discordant results of an adult male patient who was referred to sampling for serum TSH and PRL testing within 12-hour period after an intercontinental flight case to emphasize the importance of timing of sampling patient’s sleeping status for hormone testing.

Sleeping status is an important preanalytical variable when it comes to analytes related to circadian rhythm. Diurnal secretion of some analytes, mainly hormones can get affected by any sleep disturbance. Both PRL and TSH have been shown to have diurnal variations. In the literature, it has been shown that PRL has a diurnal variation with a bimodal pattern (*4*). Concentrations of prolactin in serum are known to be elevated during the night in men (*5*). In a recent study, diurnal variation values of TSH were given as 10.6 to 32.2% (*6*). Long-distance, intercontinental flights may lead to a sleep disturbance called jetlag causing altered metabolic status due to disruption of dark-light and sleep-wake cycles (*4*, *6*).

Jetlag (flight dysrhythmia) is a condition affecting individuals flying across six or more time zones. Jetlag mainly disturbs the mechanisms regulating the circadian rhythmicity. A disturbed dark-light cycle can alter the hormonal balance of the organism. Hormones of CNS which are released from the hypothalamus, pineal and pituitary glands can be significantly affected by jet-lag. The main controller of circadian rhythm in humans is located in suprachiasmatic nuclei (SCN) of the hypothalamus (*3*). Light exposure is perceived by cells of SCN *via* retinohypothalamic tract and SCN regulates the secretion of CRH (corticotropin-releasing hormone) and food intake. Signals from SCN also activates the secretion of melatonin. Melatonin hormone secreted by the pineal gland during darkness is one of the components of a biological clock. Melatonin also acts on the hypothalamus. Light and melatonin act on the hypothalamus regulating hormones, mood, mental performance, and immune system.

One of the main hormones secreted by the hypothalamus is thyrotropin-releasing hormone (TRH) and affects both TSH and PRL secretions from the anterior pituitary gland. Jetlag alters the secretion of TRH, acting indirectly on TSH and PRL concentrations to elevate (*3*). Our data is consistent with this information.

It has been known that it takes generally 4 to 6 days after travelling for individuals who fly over six or more different time zones to recover jetlag without any intervention (*2*). Our case is consistent with this pattern.

Jet-lag symptoms may be more severe if travelled from West to East (eastward) rather than East to West (westward) (*7*). Our case history seems to be parallel to this information as the flight was from the USA to Turkey (eastward).

## What you should do in your laboratory to prevent such errors

The only sleep disturbance related to travel history is jet-lag. In cases of jet-lag, sleeping status and travel history become the two most important preanalytical variables. Even though it is a rare situation and one cannot imagine anyone coming directly after an intercontinental flight to a laboratory, anything is possible in medical practice and we as laboratory professionals have to be cautious about “jet-lagged” patients.
